# Effect of polyacrylic acid pretreatment before CPP-ACP application in remineralization of demineralized root dentin: An in vitro study

**DOI:** 10.34172/joddd.025.43997

**Published:** 2025-12-31

**Authors:** Arezoo Ghasemi, Elmira Jafari Navimipour, Soodabeh Kimyai, Parnian Alizadeh Oskuee, Mehdi Daneshpooy, Ziba Islambulchilar, Sevda Riyahifar

**Affiliations:** ^1^Department of Operative Dentistry, School of Dentistry, Tabriz University of Medical Sciences, Tabriz, Iran; ^2^Department of Pharmaceutics, Faculty of Pharmacy, Tabriz University of Medical Sciences, Tabriz, Iran; ^3^Department of Biostatistics, School of Paramedical Sciences, Shahid Beheshti University of Medical Sciences, Tehran, Iran

**Keywords:** CPP-ACP, Dentin, Polyacrylic acid, Remineralization

## Abstract

**Background.:**

This study evaluated the efficacy of polyacrylic acid (PAA) pretreatment before application of casein phosphopeptide–amorphous calcium phosphate (CPP-ACP) in remineralizing demineralized root dentin.

**Methods.:**

Thirty-nine dentin specimens from extracted human third molars were prepared. All the specimens were standardized for Vickers microhardness at baseline (VHNba). Then the specimens were randomly divided into three groups (n=13): (1) untreated demineralized dentin, (2) CPP-ACP treatment, and (3) PAA pretreatment before CPP-ACP application. All the specimens were subjected to 72 hours of demineralization. Subsequently, treatments were administered according to group protocols and a 28-day pH-cycling regimen. Then, Vickers microhardness (VHN) was assessed after demineralization (VHNde) and after treatment (VHNre). The difference between VHNre and VHNde was calculated as ΔVHN and compared between the groups. Data were analyzed using one-way ANOVA, repeated-measures ANOVA, and post hoc Bonferroni tests (P<0.05).

**Results.:**

Demineralization significantly decreased dentin microhardness in all the groups (*P*<0.001). In contrast, microhardness values increased significantly after applying the treatment protocol (*P*<0.001). Specimens that received PAA pretreatment before CPP-ACP application showed a considerably higher ΔVHN than those treated with CPP-ACP alone (*P*<0.0001). However, neither group returned to the initial values.

**Conclusion.:**

Applying CPP-ACP, either alone or after pretreatment with PAA, increased the microhardness of demineralized root dentin and promoted remineralization. Pretreatment with PAA enhanced this remineralization effect, suggesting its potential use in conservative dentin treatment strategies.

## Graphical Abstract

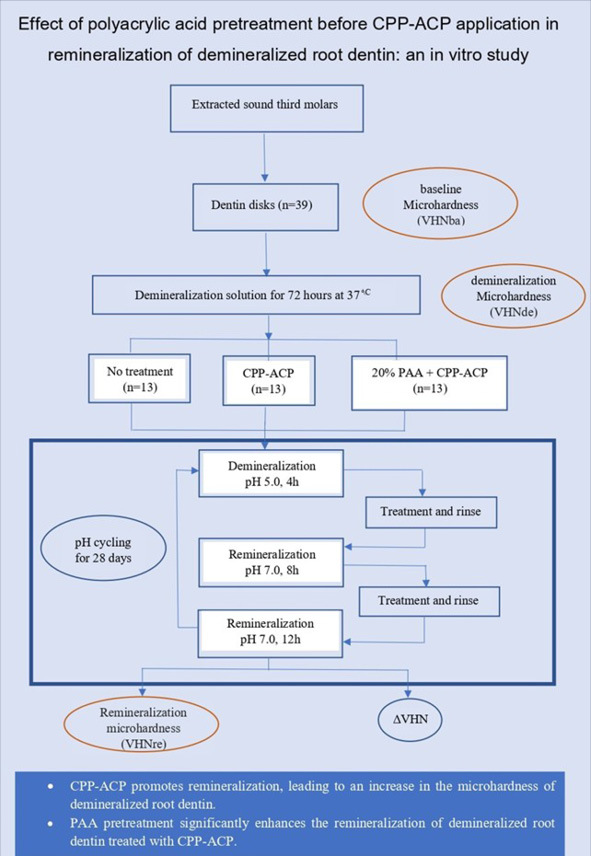

## Introduction

 Root caries is a widespread issue affecting a significant percentage of the elderly population. Contributing factors include increased lifespan, a higher number of retained teeth, gingival recession exposing root surfaces, xerostomia induced by medications for chronic systemic diseases, and head and neck cancer radiotherapy.^1,2^

 Caries develops when demineralization outweighs remineralization.^3^ Demineralization leads to the loss of minerals from dental hard tissues. This process begins when the oral pH drops below the critical thresholds of 5.5 for enamel and 6.2 for dentin, causing hydroxyapatite to dissolve and release calcium (Ca^2+^) and phosphate (PO₄^3-^) ions into the oral environment.^4^ Since dentin’s critical pH is higher than the enamel’s, exposed root dentin demineralizes more easily even in mildly acidic conditions, making root caries progress at approximately twice the rate of coronal caries.^2^ Moreover, acid exposure reveals type I collagen in dentin, which is then denatured and degraded by host and bacterial matrix metalloproteinases (MMPs) and other bacterial proteases, weakening its structural integrity and resulting in dentin cavitation.^5^

 Understanding the balance between demineralization and remineralization processes is key to caries management. Promoting remineralization is recommended for root surface lesions where the surface integrity of the exposed root has not been compromised, and protective factors, including remineralizing agents, are available to restore lost minerals to the demineralized dentin matrix.^5^

 Two main dentin remineralization strategies exist. The first one is the classical top-down ionic theory, which involves the diffusion of calcium and phosphate ions, often from saliva or therapeutic agents, into the demineralized dentin structure, where they precipitate and rebuild the mineral lattice. It relies on residual hydroxyapatite crystals as nucleation centers for crystal growth. The second strategy is the biomimetic bottom-up approach in which collagen fibrils provide an organic scaffold for mineral deposition, under the control of non-collagenous proteins (NCPs).^6^ NCPs regulate natural dentin mineralization, guiding calcium and phosphorus deposition onto collagen fibrils to form hydroxyapatite.^3^ Unlike classical methods, biomimetic dentin remineralization does not require residual hydroxyapatite crystals in partially demineralized dentin. Instead, it relies on the infiltration of fluid-like ACP (amorphous calcium phosphate) particles into collagen fibrils, where they precipitate and form new crystals within the fibrils, promoting intrafibrillar mineralization.^3,6-8^

 Current remineralizing agents, such as fluoride, have limitations in remineralizing dentin. Although high-concentration fluoride is often recommended to prevent root caries in older adults, its effectiveness depends on the natural availability of calcium and phosphate in saliva. When the quantity or quality of saliva declines, as commonly seen in elderly or irradiated patients, the risk of dental caries increases because fewer minerals are available to support remineralization. Additionally, while silver diamine fluoride is effective, its use on permanent teeth is limited due to its unfavorable staining effects.^9^

 One of the remineralizing agents currently used is CPP-ACP, which provides bioavailable calcium and phosphate ions. It is well studied in enamel, but less in dentin.^7,10^ Compared to enamel, remineralizing dentin is more challenging. CPP-ACP shows variable effectiveness in dentin due to its heterogeneous structure and limited residual hydroxyapatite content, underscoring the need for adjunctive strategies. One of these strategies can be PAA pretreatment of dentin before CPP-ACP application.^3,7,11^ PAA, as an analogue of NCPs, can be used to stabilize ACP and prevent the aggregation of ACP fluid nanoparticles into larger particles and their conversion to apatite before entering collagen fibers.^3,12^ On the other hand, PAA, as a conditioner, can remove the smear layer from the dentin surface, making hydroxyapatite directly accessible to interact with the subsequently used material.^13^

 Since no studies have so far investigated the role of PAA pretreatment before CPP-ACP application in remineralization of artificially demineralized root dentin, the present in vitro study aimed to evaluate this subject by measuring dentin microhardness. Two null hypotheses were tested: (1) Application of the treatment protocols does not change the VHNre compared with the VHNde. (2) There is no difference in ΔVHN between dentin pretreated with PAA before applying CPP-ACP and dentin treated with CPP-ACP alone.

## Methods

###  Sample Preparation

 Thirty-nine sound human third molars, extracted for impaction or periodontal reasons, were collected from the Oral and Maxillofacial Surgery Department of Tabriz Dental School. This study was approved by the Ethics Committee of Tabriz University of Medical Sciences (IR.TBZMED.DENTISTRY.REC.1403.020).

 The minimum required sample size for this study was calculated at 39 specimens (n = 13 per group), based on a power of 0.80, a type I error rate of 0.05, an effect size of 0.630, and an anticipated dropout rate of 20%. The calculation was performed using PASS software, version 20.0.6.^1^

 The teeth were examined visually and with a dental explorer to exclude any with caries, fractures, cracks, restorations, hypoplasia, or wear. Afterwards, they were cleaned and stored continuously in deionized water containing 0.1% thymol at room temperature for up to one month until use. Then the crowns were sectioned at the cementoenamel junction (CEJ) using a low-speed diamond saw (Isomet, Buehler Ltd., Lake Bluff, IL, USA) under water coolant. The roots were split buccolingually, and 4 × 4 × 1 mm dentin specimens were prepared from the buccal and lingual mid-root regions. Buccally and lingually prepared specimens were equally distributed among the groups to minimize surface variability. All the specimens were polished using 600-, 800-, and 1200-grit silicon carbide papers (Siawat WA, Switzerland), rinsed in deionized water, and embedded in acrylic resin (Acropars, Marlic Co., Tehran, Iran), with the dentin surface facing upward.

###  Baseline Microhardness Measurements

 VHNba was measured using a Vickers microhardness tester (Micromet II, Buehler Ltd., Lake Bluff, IL, USA) with a 0.98-N load applied for 15 seconds at three similar positions, 500 µm apart. The average value was recorded. The VHNba values of the samples were subjected to one-way ANOVA with a significance level of *P* < 0.05. After confirming that there was no statistically significant difference between the three groups (*P* = 0.49), they were included in the study.^1,10^

 Subsequently, the samples were randomly divided into three groups (n = 13):

- *Group 1:* demineralized dentin without treatment
- *Group 2:* demineralized dentin treated with CPP-ACP
- *Group 3:* demineralized dentin pretreated with 20% PAA followed by CPP-ACP treatment

###  Demineralization and Remineralization Solutions

 The demineralization solution was 0.05-M acetic acid containing 2.2-mM CaCl_2_·2H_2_O and 2.20-mM KH_2_PO_4_ (Kimia Shimi Company, Tabriz, Iran), and was adjusted to pH = 5.0.^1^

 The remineralization solution contained 1.5-mM CaCl_2_·2H_2_O, 0.90-mM KH_2_PO_4_, and 130-mM KCl (Kimia Shimi Company, Tabriz, Iran), and was adjusted to pH = 7.0.^1^

###  Preparation of Artificial Lesions

 All the specimens were immersed in a demineralization solution at 37°C for 72 hours. VHNde was then measured considering the same conditions as previously described for baseline measurement.

###  Experimental Procedures

 The demineralized dentin disks were subjected to the treatment protocol specified for each group and to pH cycling for 28 days, during which they were placed in the demineralization solution for 4 hours each day, then washed and subjected to the treatment regimen. After washing, the samples were placed in the remineralization solution for 8 hours. The treatment was then repeated, and after washing, they were placed in the remineralization solution for an additional 12 hours. All the samples were placed in containers containing 15 mL of solution, and all the solutions were freshly prepared before use. To standardize treatment, the entire process was handled by a single person.

 The treatment protocol in the study groups was as follows:

####  Group 1: Demineralized Dentin without Treatment

 In this group, no specific treatment was performed; the samples were simply exposed to pH cycling.

####  Group 2: Demineralized Dentin Treated with CPP-ACP

 In this group, treatment with CPP-ACP was administered twice daily. For this purpose, the samples were covered with CPP-ACP (GC Tooth Mousse; GC America Inc.; USA) for 3 minutes, then washed with deionized water, and placed in the pH cycler.

####  Group 3: Demineralized Dentin Treated with CPP-ACP after Pretreatment with PAA

 In this group, the samples were treated with 20% PAA (Cavity Conditioner; GC America Inc.; USA) for 10 seconds, then washed with deionized water, and subsequently treated with CPP-ACP as in group 2. Treatment in this group was also performed twice a day. After the specified time and washing with deionized water, the samples were placed in the pH cycler.

 At the end, VHNre was measured for all the groups. Then the values were compared within each group at three stages. Also, the difference in microhardness values after demineralization and following treatment was calculated (∆VHN = VHNre - VHNde), and compared between the groups.

###  Statistical Analysis

 The study data were analyzed using IBM SPSS v26.0. Descriptive statistics were reported using mean ± standard error of the mean (SEM). Given the normal distribution of the data, as assessed by the Shapiro-Wilk test, one-way ANOVA and repeated-measures ANOVA were used to analyze the data. Also, post hoc Bonferroni tests were used to compare the groups. A significance level of P < 0.05 was considered.

## Results

Table 1 presents the descriptive statistics of the mean differences in microhardness values at three different stages for each group.

**Table 1:** Microhardness values for each group at different stages

**Group**	**Stage**	**Mean±Std. error**	**95% confidence interval**	
			**Lower bound**	**Upper bound**
No treatment (control)	VHNba	57.651 ± 1.419	54.774	60.529
	VHNde	14.331 ± 0.515	13.287	15.374
	VHNre	17.877 ± 0.508	16.846	18.908
CPP-ACP	VHNba	60.323 ± 1.419	57.445	63.201
	VHNde	14.882 ± 0.515	13.839	15.926
	VHNre	19.313 ± 0.508	18.282	20.344
PAA + CPP-ACP	VHNba	59.892 ± 1.419	57.015	62.770
	VHNde	15.282 ± 0.515	14.239	16.326
	VHNre	20.795 ± 0.508	19.764	21.826

*Note:* ACP: amorphous calcium phosphate, CPP-ACP: casein phosphopeptide–amorphous calcium phosphate, PAA: polyacrylic acid, VHNba: Vickers microhardness at baseline, VHNde: Vickers microhardness after demineralization, VHNre: Vickers microhardness after treatment.

 The results of the repeated-measures ANOVA indicated a significant main effect of stage on the dependent variable, with substantial differences observed across stages (*P* < 0.001). This suggests a strong effect of stage. In contrast, the stage × group interaction was not statistically significant (*P* = 0.460), indicating that the pattern of change across stages did not differ significantly between the groups.

 A pairwise comparison of groups using post hoc Bonferroni tests showed that VHNde decreased significantly compared to VHNba. Also, after treatment, a significant increase in the VHNre was observed, but it did not reach baseline levels (Table 2).

**Table 2:** Overall comparison of VHN at different stages

**Stage**	**Mean±Std. error**	**95% confidence interval**		**Significance** ***P*** **<0.05 **^a^
		**Lower bound**	**Upper bound**	
VHNba	59.289 ± 0.819	57.627	60.950	AB
VHNde	14.832 ± 0.297	14.229	15.434	AC
VHNre	19.328 ± 0.294	18.733	19.924	BC

^a^ Similar letters (A, B, C) indicate statistical significance for VHN between different stages. *Note:* VHNba: Vickers microhardness at baseline, VHNde: Vickers microhardness after demineralization, VHNre: Vickers microhardness after treatment.

 To compare ∆VHN values between the groups, one-way ANOVA was used. The results showed significant differences between the groups (*P* < 0.0001). Subsequently, pairwise comparisons between groups were performed using the Bonferroni method (Table 3).

**Table 3:** Descriptive statistics of ∆VHN values in the study groups

**Group**	**Mean±Std. deviation**	**95% confidence interval**		**Significance** ***P*** **<0.05 **^a^
		**Upper bound**	**Lower bound**	
No treatment (control)	3.546 ± 0.2065	3.671	3.421	AB
CPP-ACP	4.431 ± 0.1557	4.525	4.337	AC
PAA + CPP-ACP	5.512 ± 0.1421	5.597	5.426	BC

^a^ Similar letters (A, B, C) indicate statistical significance for ∆VHN between different groups. *Note:* ACP: amorphous calcium phosphate, CPP-ACP: casein phosphopeptide–amorphous calcium phosphate, PAA: polyacrylic acid.

**Figure 1:**
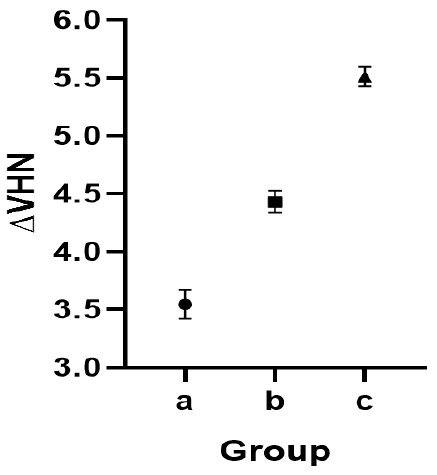
∆VHN values in the study groups. A) No Treatment. B) CPP-ACP. C) PAA + CPP-ACP. *Note:* ACP: amorphous calcium phosphate, CPP-ACP: casein phosphopeptide–amorphous calcium phosphate, PAA: polyacrylic acid.

 According to Table 3 and Figure 1, the difference between every pair of groups was statistically significant (*P* < 0.0001). In other words, in both intervention groups, microhardness increased significantly compared to the control group. Furthermore, the increase in microhardness following PAA pretreatment was significantly greater than that observed with CPP-ACP alone.

## Discussion

 Traditionally, the management of carious teeth involves the removal of decayed tissue followed by restoration. With the rise of minimally invasive and protective dentistry, preserving non-infected demineralized tissue and remineralizing it using bioactive remineralizing agents is now favored. This approach supports pulpal health, limits structural loss, and increases tooth longevity. However, dentin remineralization remains a challenging issue, according to recent studies.^3^

 Since dentin microhardness directly reflects its mineral content, we used this metric to assess the extent of remineralization.^3,10^ We used root dentin specimens with similar VHNba to ensure the validity of comparisons. Subsequently, we created artificial carious lesions by exposing all the specimens to a demineralizing solution at pH = 5 for 72 hours. Then VHNde was measured. The results showed a significant decrease in microhardness across all the groups after demineralization, consistent with the acidic conditions below the dentin’s critical pH. Similarly, Wu et al.^1^ demonstrated that this solution effectively demineralizes dentin specimens.

 To simulate oral conditions, a pH-cycling method was used in our study. This in vitro protocol exposes specimens to alternating demineralization and remineralization, mimicking the dynamic changes in mineral saturation of tooth structure during natural caries progression. Acidic phases simulate destructive effects on superficial and subsuperficial dental layers. In contrast, remineralization phases use neutral calcium and phosphate-containing solutions to mimic the protective effects of saliva or other remineralizing agents. However, a limitation of pH-cycling models is that they do not evaluate the antimicrobial effects of anticariogenic agents.^3,14^

 After demineralization, we treated the specimens according to our protocol. Treatment with CPP-ACP alone, or after pretreatment with PAA, significantly increased VHNre in the demineralized dentin. As a result, we rejected our first null hypothesis. Additionally, pretreatment with PAA before the application of CPP-ACP led to a greater increase in ∆VHN compared to using CPP-ACP alone, prompting us to reject our second null hypothesis as well.

 CPP-ACP is a well-known bioactive remineralizing agent. CPP is a peptide derived from milk casein, exhibiting excellent biocompatibility and cell binding. Reis et al.^15^ and Aboulfotoh et al.^16^ reported CPP-ACP as an effective dentin remineralizing agent consistent with our findings. Heshmat et al.^10^ advocated its use on demineralized dentin lesions. According to these studies, the effectiveness of CPP-ACP for dentin remineralization was due to the controlled release of calcium and phosphate ions from the CPP-ACP paste. Since CPP prevents calcium phosphate crystal growth, Ca-P likely forms a stable nano-composite with CPP. CPP-ACP facilitates mineral delivery by binding to demineralized areas of teeth and gradually releasing Ca^2+^ and PO₄^3-^ to prevent demineralization and promote dentin remineralization.^3,7,11^ Studies also suggest that CPP-ACP acts as a buffer, mitigating acidic damage.^11^ Poggio et al.^17^ observed significant protective effects of CPP-ACP against enamel and dentin demineralization, using atomic force and scanning electron microscopy, although the effect was greater on enamel. Dentin remineralization by CPP-ACP alone remains challenging due to weaker intrafibrillar mineralization; as a result, in recent years, many efforts have been made to increase this effect.^7^

 In the present study, we used PAA as a pretreatment agent, and it increased the CPP-ACP effect and subsequent dentin remineralization. Cao et al.^11^ demonstrated that using PAA enhances intrafibrillar and interfibrillar remineralization and improves ACP stability. Fraga et al.^18^ similarly showed PAA’s efficacy in dentin mineralization. Also, Qi et al.^19^ used PAA as an ACP stabilizer for biomimetic collagen remineralization with positive outcomes. To explain this effect, it can be noted that PAA is one of the NCP analogs that mimics calcium phosphate binding sites in dentin matrix proteins like DMP1 and stabilizes ACP. It prevents the aggregation of ACP nanoparticles into larger particles before entering the collagen fibers, and facilitates the infiltration of fluidic ACP precursors deep into the collagen matrix.^3^ On the other hand, PAA, as a pretreatment agent, is a conditioner that can remove the smear layer from the dentin surface, making the hydroxyapatite directly in contact with the subsequently used material, which is CPP-ACP here. Therefore, it appears to enhance CPP-ACP penetration into deeper dentin. Moreover, a slight dentin demineralization occurs after PAA application, and a submicron interdiffusion layer is formed. The residual hydroxyapatite within the demineralized collagen fibrils may also serve as receptors for additional chemical reactions.^13^

 In the present study, despite significant microhardness recovery following PAA pretreatment and CPP-ACP application, baseline hardness was not restored. Similar to the present study, Manzoor et al.^20^ showed that CPP-ACP treatment over 28 days improved dentin microhardness after demineralization, though not to baseline levels. To increase the validity of the results, the same issue should be repeated with a larger sample size and over a longer period. Also, it is suggested that microscopic studies and other laboratory tests be used to interpret the results more accurately.

 In the present study, we used PAA and CPP-ACP as directed by the manufacturers’ instructions. As mentioned in the GC Tooth Mousse brochure, there are two methods for applying this material: in-office and at-home. Therefore, to pretreat dentin with PAA in the clinic, we can use this material in-office under a dentist’s supervision. However, for at-home applications, there are limitations to using PAA, and a need for new products is felt. Difficulties in controlling the contact time of the materials, rapid removal of the agents by salivary flow, and variations in patient compliance and acceptance should be considered.

 Considering the limitations of the current in vitro study, it seems that PAA pretreatment enhances CPP-ACP remineralization effects on demineralized root dentin, offering promising strategies to prevent root surface caries, especially in susceptible patients; however, the results of this in vitro study should be generalized to the clinic with caution. Oral environment factors, such as the presence of oral biofluids, numerous microorganisms, and pH fluctuations, can influence the remineralization efficacy of these materials. Also, other limitations in these laboratory studies include the absence of the natural biological process of dental caries development and the lack of pulpal fluid effect, which can affect the results in real conditions. Therefore, similar clinical studies should be designed to investigate the results in vivo.

## Conclusion

 Within the limitations of this study, PAA, as a pretreatment material, significantly enhanced the effect of CPP-ACP in the remineralization of demineralized root dentin. While these improvements were statistically significant, their clinical relevance should be validated through further in vivo studies.

## Competing Interests

 The authors declare that they have no conflict of interest.

## Ethical Approval

 This study was approved by the Ethics Committee of Tabriz University of Medical Sciences (IR.TBZMED.DENTISTRY.REC.1403.020).
